# A metrological approach to the analysis of choroidal thickness by optical coherence tomography 3D scans in myopia research

**DOI:** 10.1038/s41598-019-56915-9

**Published:** 2019-12-30

**Authors:** Katharina Breher, Arne Ohlendorf, Siegfried Wahl

**Affiliations:** 1 Institute for Ophthalmic Research, Eberhard Karls University Tuebingen, Tuebingen, 72076 Germany; 2 Carl Zeiss Vision International GmbH, Aalen, 73430 Germany

**Keywords:** Translational research, Translational research

## Abstract

In myopia research, changes of choroidal thickness in response to optically induced signals serve as predictor for changes in axial length that might be correlated with myopia progression. Optical coherence tomography (OCT) provides a tool for imaging the choroid, however, with certain difficulties because of a limited visibility of the scleral-choroidal interface. Considering the previously reported effect sizes of thickness change in human myopia research, this study investigated the repeatability of automated 3D choroidal segmentation across the macular area of 6 × 6 mm^2^. Fifteen subjects underwent nine volume scans in two OCT devices with analysis of the 95% interval of repeatability, intersubject and intrasubject variations, as well as interdevice agreement. Repeatability generally improved with increasing eccentricity from the fovea. The nasal perifoveal region exhibited the best repeatability with ±19 and ±21 *μ*m in both OCT devices, whereas the subfovea showed a repeatability of ±57 and ±44 *μ*m, respectively. High inter- and intrasubject variations were observed, together with a negative bias in the device agreement. Although there is still limited data on thickness changes of the nasal choroid, future studies could focus more on measuring the effect size in the nasal perifoveal area to account for metrological issues in choroidal segmentation.

## Introduction

Optical coherence tomography (OCT) allows *in-vivo* imaging of retinal structures. Especially imaging the choroid has become of great interest in the field of myopia research^1^. Previous studies in animals^2–4^ and humans^5–8^ have shown that the choroid might be capable to change its thickness in a bi-directional fashion in response and in relation to the sign of defocus already after a short period of time and in anti-phase to the axial length. Moreover, physiological^9–14^ and defocus-manipulated circadian thickness changes^15,16^ of the choroid gained more interest together with differences in the absolute thickness and distribution patterns of choroidal thickness between myopes and emmetropes^17–19^. The aforementioned choroidal reaction, rhythm and global distribution therefore might serve as a predictive biomarker for future axial length development.

However, measuring choroidal thickness changes from OCT images can cause difficulties due to a limited visibility of the choroidal-scleral interface, which is dependent on the absolute choroidal thickness and the pigmentation of the retinal pigment epithelium in healthy eyes^20,21^. Measurements of choroidal thickness can be performed manually, with semi-automated as well as fully automated segmentation algorithms. Previous studies have investigated the repeatability, correlation and agreement of manual measurements of choroidal thickness by one or more examiners or with different spectral-domain OCT devices^21–26^. In addition, various algorithms for (semi-)automated choroidal segmentation have been developed, validated and compared to the manual thickness evaluations, which served as gold standard^27–30^. These studies report good correlations of choroidal measurements of approximately 20 *μ*m. However, they were mainly focused on the subfoveal choroid, with only few of them reporting results from single measurement points outside of the subfoveal region, which were obtained from line scans. Furthermore, the eventual interpretation of the overall reliability of choroidal thickness segmentation and analysis is dependent on the usage of these results with the associated effect sizes. For example, the aforementioned studies in the field of human myopia research have reported choroidal thickness changes of 10 *μ*m or even less, whereas clinical applications for choroidal pathologies generally present with considerably higher changes of more than 100 *μ*m^31–33^, and are therefore less sensitive to metrological influences by the OCT devices and analysis methods.

Thus, the aim of the current study is to evaluate the repeatability and agreement of a published open-source automated segmentation algorithm for OCT volume scans^34^ in two different OCT devices (ZEISS CIRRUS HD-OCT 5000, Carl Zeiss Meditec Inc., Dublin, CA, USA and HRA + OCT SPECTRALIS, Heidelberg Engineering, Germany) across the macular area using volume scans instead of multiple line scans. The overall purpose is to critically define the reliability of OCT measurements in regards to the effect size of choroidal thickness changes/differences with a maximum of 20–30 *μ*m as they have been reported in human myopia research^5–14,17–19,35,36^. Another aim of the study is to particularly investigate regional differences in the repeatability of choroidal segmentation, in order to give meaningful recommendations for future measurement locations, such that the repeatability of the measurement location itself does not doubt the found effect sizes.

## Results

### Subjects

**Figure 1:**
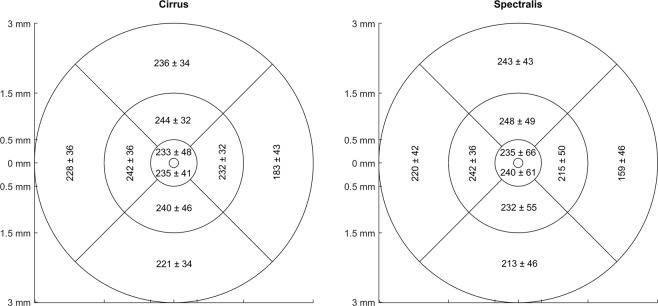
Choroidal thickness (in microns) across the different ETDRS areas averaged over all subjects for the ZEISS Cirrus OCT and the Spectralis HRA + OCT.

Spherical equivalent refractive errors from objective refraction of the right eye ranged from −0.50 D to −6.25 D with a mean of −3.18 D ± 2.01 D. Mean choroidal thicknesses in the different Early Treatment of Diabetic Retinopathy Study (ETDRS)^37^ areas are displayed in Fig. 1. Generally, the choroidal thickness decreases towards the periphery of the macula. It moreover varies among the ETDRS regions with the nasal area exhibiting the thinnest choroids between 159 ± 46 *μ*m and 232 ± 32 *μ*m, followed by, in increasing order, the inferior regions, the central, temporal and superior areas, that show an almost 100 *μ*m thicker choroid with up to 248 ± 49 *μ*m.

### Repeatability in the macular area

**Figure 2:**
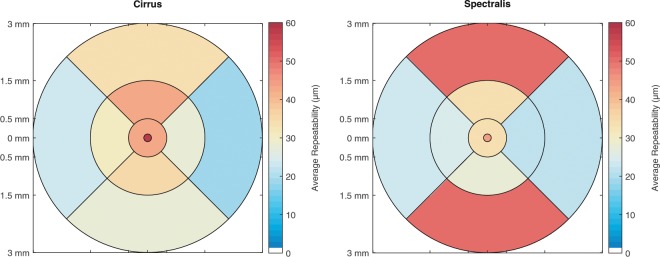
Colour-coded repeatability in the different ETDRS areas of the right eye as measured with both OCT devices.

The repeatability of the analysis of choroidal thickness varies among the different ETDRS areas of the macula in both devices as seen in Table 1. Generally, the repeatability improves with increasing distance (eccentricity) from the fovea for all ETDRS areas measured with the ZEISS Cirrus, and for all ETDRS except the nasal section when measured with the Heidelberg Spectralis OCT. The repeatability of measurements in the subfoveal region is among the highest values for both devices with 57 *μ*m and 44 *μ*m, whereas the nasal region, especially at the diameter of 6 mm exhibits the best repeatability with 19 *μ*m and 22 *μ*m for both OCT devices. Figure 2 shows the repeatability values from Table 1 in a colour-coded map across the macular area.

**Table 1:** Repeatability values with 2.5% and 97.5% limits of the reference intervals of the different ETDRS sections. Repeatability is defined as half the length of the reference interval.

	ZEISS Cirrus			Heidelberg Spectralis		
	Repeatability	2.5%	97.5%	Repeatability	2.5%	97.5%
Subfoveal	±57	−59	55	±44	−43	45
Central 1 mm	±43	−42	44	±34	−34	34
Inferior 3 mm	±35	−36	34	±28	−29	27
Superior 3 mm	±42	−43	42	±34	−36	33
Nasal 3 mm	±27	−26	27	±22	−22	23
Temporal 3 mm	±31	−33	29	±24	−23	25
Inferior 6 mm	±27	−28	27	±50	−77	22
Superior 6 mm	±34	−32	35	±50	−64	35
Nasal 6 mm	±19	−19	18	±21	−22	20
Temporal 6 mm	±23	−23	24	±23	−23	23

### Intersubject and intrasubject variability

**Figure 3:**
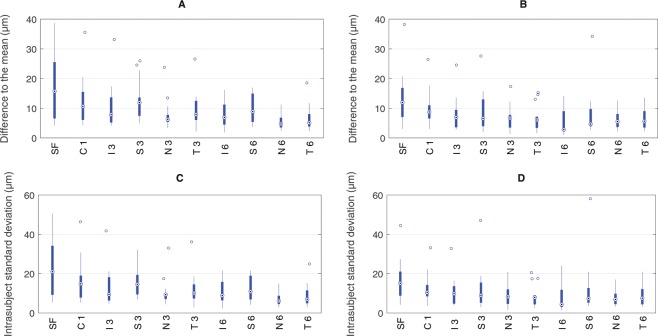
Difference to the mean from all subjects across the different ETDRS regions to describe the variability between subjects for ZEISS Cirrus (**A**) and Heidelberg Spectralis (**B**). Standard deviation of the raw choroidal thickness measurements from all subjects across the ETDRS regions to describe the variability within one subject for ZEISS Cirrus (**C**) and Heidelberg Spectralis (**D**). Note that the abbreviations of the ETDRS areas on the x-axis are composed of the specific region and its diameter in mm.

The repeatability varied greatly between the individual subjects as can be seen on Fig. 3 in the top two plots A and B. The average difference to the mean - not to be confused with the 95% reference interval derived from the cumulative distribution function (CDF) as described in the methods section and reported in Table 1 - for all subjects in the subfoveal region was 17 ± 11 *μ*m and 13 ± 9 *μ*m for both OCT devices, respectively. However, the individual differences ranged from 4 *μ*m to 39 *μ*m (ZEISS Cirrus) and from 3 *μ*m to 38 *μ*m (Heidelberg Spectralis). In contrast, the perifoveal nasal ETDRS section showed generally lower averaged differences to the mean with 6 ± 3 *μ*m with a range of 2 *μ*m to 13 *μ*m for both OCT devices.

However, analysis of choroidal thickness did not only vary between subjects but also within the single subjects. If the intrasubject standard deviation was high, this indicated that the choroidal thickness analysis in this subject led to very different results. Figure 3 gives an overview about the standard deviation of the differences to be mean across the study population in the bottom row plots C and D. Exemplarly, the subfoveal region showed the highest range of intrasubject variability from 5 *μ*m and 4 *μ*m up to 51 *μ*m and 44 *μ*m, for each of the OCT devices. In contrast, the outermost nasal section exhibited the least standard deviations for single subjects with on average 7 ± 4 *μ*m measured by ZEISS Cirrus, and 8 ± 4 *μ*m by Heidelberg Spectralis OCT.

### Agreement between both devices with automated choroidal segmentation

**Figure 4:**
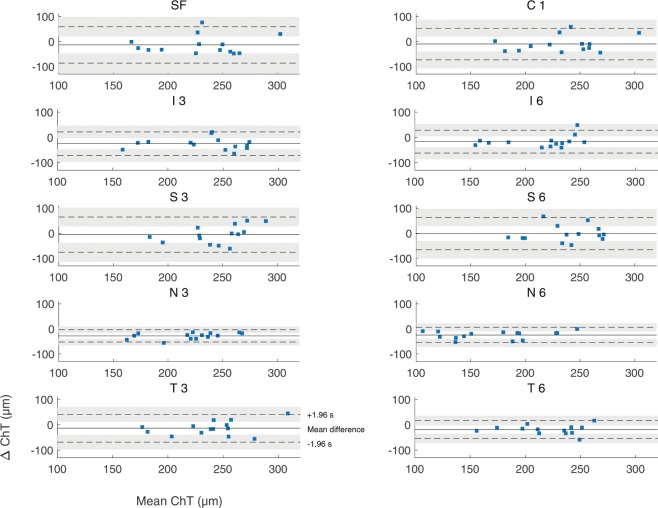
Bland-Altman plots for comparison of choroidal thickness analysis between both OCT devices for the single ETDRS areas. The x-axes of the plots show the mean of ChT measurements in both devices, the y-axes the difference between them. The subfoveal and superior regions show the most spreaded intervals of agreement in contrast to the nasal areas.

Bland–Altman analysis^38^ and Intraclass correlation (ICC)^39^ coefficients were used for statistical analysis of the agreement of choroidal thickness measurements between both devices. Results are shown in Table 2 (limits of agreement), Table 3 (ICC) and Fig. 4 (Bland-Altman plots). It is noteworthy that the choroidal thickness analysis yielded constantly higher thickness values for the ZEISS Cirrus than for the Heidelberg Spectralis, which is indicated by the negative bias of the mean differences in Table 2. The superior regions showed here the least bias but widely spread limits of agreement in contrast to the nasal areas with the highest mean difference but smallest limits of agreement. However, for all other ETDRS areas, the limits of agreement and their associated 95% confidence intervals become smaller with increasing retinal eccentricity. This relationship is confirmed by the ICC coefficients in Table 3.

**Table 2:** Mean differences, limits of agreement (LoA) and their 95% confidence intervals (CI) for each retinal area. Negative mean differences mean that the choroidal thickness measurements obtained by the ZEISS Cirrus OCT were thicker than these obtained by the Heidelberg Spectralis OCT.

	MD (*μ*m)	95% LoA (*μ*m)	95% CI upper limit (*μ*m)	95% CI lower limit (*μ*m)
**Heidelberg Spectralis vs**. **ZEISS Cirrus**				
Subfoveal	−14	±73	20 to 98	−125 to −48
Central 1 mm	−10	±63	20 to 87	−107 to −39
Inferior 3 mm	−25	±47	−3 to 46	−96 to −47
Superior 3 mm	−5	±60	28 to 103	−113 to −38
Nasal 3 mm	−28	±25	−16 to 10	−66 to −39
Temporal 3 mm	−13	±55	12 to 71	−98 to −39
Inferior 6 mm	−17	±45	4 to 52	−86 to −38
Superior 6 mm	−1	±64	29 to 97	−100 to −31
Nasal 6 mm	−24	±31	−9 to 23	−71 to −39
Temporal 6 mm	−19	±35	−2 to 36	−73 to −35

**Table 3:** ICC coefficients with 95% confidence intervals for the retinal areas. The nasal areas show excellent correlations compared to the subfoveal and superior sections with the lowest coefficients but still good overall correlation.

	ICC	95% CI	
		Lower	Upper
Subfoveal	0.773	0.29	0.93
Central 1 mm	0.804	0.39	0.94
Inferior 3 mm	0.902	0.69	0.97
Superior 3 mm	0.633	−0.14	0.88
Nasal 3 mm	0.965	0.89	0.99
Temporal 3 mm	0.842	0.51	0.95
Inferior 6 mm	0.884	0.64	0.96
Superior 6 mm	0.682	0.01	0.90
Nasal 6 mm	0.968	0.90	0.99
Temporal 6 mm	0.916	0.74	0.97

## Discussion

The current study evaluated the overall repeatability, intersubject and intrasubject repeatability, as well as agreement of choroidal thickness measurements in two OCTs across the macula. To our best knowledge, this is the first study that analyzed choroidal thickness with automated segmentation^34^ and further splitted into the different ETDRS regions for separate analysis. The results showed consistently better repeatability in the nasal section compared to the other measured areas, especially in the case of the central and subfoveal divisions. One obvious reason for the better repeatability nasally could present the thinner choroidal thickness in that area, which allows better scan depth into the tissue, thus clearer imaging of the choroidal-scleral interface and therefore more reliable segmentation. However, this reasoning stands in contradiction to the worse repeatability of the inferior compared to the relatively thicker temporal choroid. Bland-Altman analysis also showed that the nasal quadrants indeed show the highest mean difference, however, they also show the smallest limits of agreement and the highest ICC coefficients. Together with the good repeatability value in those area indicates that the choroidal thickness might differ significantly between both OCTs in absolute numbers but still are reliable from a relative point of view. However, this absolute bias becomes less important in human myopia studies examining changes in response to e.g. optical defocus, where usually only the relative difference before and after exposure to defocus are measured with the same OCT device.

Moreover, previous studies using automated choroidal thickness analyses showed varying results in regards of repeatability. Twa *et al*.^30^ automatically analyzed line scans taken one hour apart from each other with resulting limits of agreement of 14 *μ*m. However, they did not distinguish between retinal locations for their analysis. Gupta and colleagues^29^ applied a 7-line macular volume scan protocol with 10 min breaks between the single scans. With their segmentation algorithm they measured choroidal thickness at the subfoveal region, nasally and temporally at 1.5 mm and 3 mm locations. These points represent the borders of the ETDRS sections in horizontal direction. They observed an excellent intrasession correlation with less pronounced differences between the evaluated locations. Moreover, the results indicated the highest repeatability subfoveally and nasally which worsened towards the temporal regions. These observed discrepancies might derive from different methodological approaches, as the present study averaged all the choroidal thickness measurement points within each ETDRS area in contrast to single points being assessed. Mansouri *et al*.^40^ measured the same-sized macular area and found an excellent correlation between consecutive measurements. However, they averaged the choroidal thickness across the entire scanning area, while also using a swept-source OCT compared to the spectral-domain devices that were used in the current study. Most recently, the widefield repeatability of a semi-automated algorithm was tested with multiple B-scans across a field of 45 × 55° using a widefield lens^41^. This resulted in a repeatability even lower than the axial resolution of the spectral-domain OCT, down to 2–3 *μ*m if whole quadrants (nasal, temporal inferior, superior) across the retinal areas are averaged. By evaluating the repeatability as a term of eccentricity, they found the foveal repeatability to be 27 *μ*m and improving towards the periphery down to 16 *μ*m. These results are in line with the results of the current study from a relative perspective, while the absolute numbers are not comparable due to differently sized measurement areas.

For a more direct numerical comparison between automated and manual segmentation, only the previous studies with reported coefficients of repeatability are discussed. These studies also found coefficients between 17 *μ*m and 49 *μ*m for the subfoveal region^22,42^, and between 27 *μ*m and 63 *μ*m if averaged across the macular region^43,44^. As this experiment was focused on comparisons of automated choroidal segmentation between different macular areas, rather than the comparison between manual and automated segmentation per se, the discussion part will not further cover the comparison between manual and automated segmentation for different OCT devices, techniques and parameters, which can be found elsewhere^27,28,30,45^.

It should be noted that the aforementioned studies using automated segmentation reported the repeatability in form of ICC coefficients and/or limits of agreement, while the current study described the reference interval as non-parametric alternative to the within-subject standard deviation derived from Analysis of Variance (ANOVA), which can be considered as a measure of repeatability with the according units of measurement (here *μ*m) for two or more repeated measurements^38,39^. This approach in units of measurement allows the direct metrological comparison to actually reported changes of choroidal thickness in human myopia research. Previous studies found effect sizes up to 20 *μ*m, on average around 10 *μ*m or even less in the subfoveal choroid in response to optically induced signals, while these reported effect sizes were accompanied by high standard deviations^5–8,35,36^. The same accounts for circadian rhythms and absolute thickness differences in the range of approximately 30 *μ*m^9–19^. Thus, the currently described measurement repeatability of subfoveal choroidal thickness highly exceeds the previously found effect sizes, which doubts the observed results from a metrological perspective. As a consequence, it would be more appropriate to analyze changes of choroidal thickness more preferably in the nasal para- and perifoveal regions, since it shows the best repeatability. However, only limited data is available on the choroidal reactions in these areas, especially from optical interventions. Choroidal thickness in respsonse to three weeks of Orthokeratology lens wear revealed the least amount of thickness change in the nasal area^35^ compared to temporally and subfoveally, whereas short-term multifocal contact lens wear showed the highest respsone in the nasal region regions^36^. Further research needs to be conducted to evaluate the possible advantages of analyzing the nasal or temporal retinal areas in the appropriate eccentricities from the fovea in order to detect choroidal thickness changes more reliably in regards to the repeatability of measurements.

Moreover, general statistical pitfalls by analyzing the intersubject variations in repeatability were worked out in the analysis process. Despite the advantages of using a single and concrete statistical value to express repeatability, such as coefficients of repeatability, reference intervals, ICC coefficients or limits of agreement, these approaches lack a differentiation between subjects. As already observed during the scan acquisition but also later during the analysis of the results, there is a high intersubject variation in repeatability. This means that an effect size, e.g. the change of choroidal thickness in response to myopic defocus, of 10 *μ*m can be already significant for one subject with a very good repeatability, but not for the other subject with a worse repeatability.

The current study also faces some limitations by itself. First of all, it included a relatively low number of participants for a repeatability analysis. However, the current study primarily aimed to evaluate the repeatability and especially its regional differences in volume scans across the central 6 × 6 mm mm^2^ retina. Although the absolute repeatability value might change in one or the other direction with an increased number of participants, the relative differences between the retinal areas most probably will persist, mainly because of choroidal thickness differences and thus associated visibility of the choroidal-scleral interface^20,21^. The current study also waived to consider magnification effects, which lead to increasing scan fields sizes with increasing myopic refractive error of the study participants and therefore potentially differently sized ETDRS areas. Resulting magnification effect from refractive errors between −0.5 D and −6.25 D translate to a maximum scan field difference of ±0.35 mm, which again translates to maximum ±30 pixels that might be inaccurately distributed. However, the current study did not evaluate single retinal point locations - which surely would be more affected by these magnification effects - but instead the median value of each of the ETDRS regions was calculated to increase the robustness against magnification effects of the following analysis. Moreover, the parameters for scan acquisition were set equally in both OCT devices except for the number of B-scans in the volume scan. Even though the algorithm interpolates missing image information in both cases, this inequality could have created the differences of repeatability between both devices. Moreover, to ensure equality of the image quality itself for the choroidal segmentation, only one frame per B-scan was averaged. Image averaging, also termed “automated real time averaging” (ART) for the Heidelberg Spectralis OCT, is capable to reduce the speckle noise and lower signal-to-noise ratio of the scan images^46^. Further studies are required to evaluate whether averaging more frames per B-scan would improve the overall repeatability across the macula with automated choroidal segmentation. Additionally, the current study was conducted with only one segmentation algorithm, which was originally developed for the Heidelberg Spectralis and its resulting scan image properties in regards to further image processing. Other algorithms might deliver different results than reported here. However, the current study also showed the successful implementation and usage of the algorithm for the ZEISS Cirrus OCT scans.

One definite advantage of automated algorithms is the fast evaluation without influences from human examiners. It also enables the analysis across a broader retinal area with multiple B-scan images in a volume scan, which would be too time intensive if segmented manually. However, this study showed that there is potential for improvement in the future. For example, the algorithms can be refined to notice even smaller amplitudes of changes on exact pixel level and therefore OCT resolution level during image processing and analysis of the scans for a better detection of the scleral-choroidal border. The OCT technology itself also undergoes a constant improvement: from time-domain to spectral-domain devices, later with EDI technology, to the newly introduced swept-source devices. This development is accompanied by constantly improving scan resolution and scan depth, for example with swept-source OCTs that provide an approximately three times higher scan depth and therefore a more complete visualization of the choroid. This progress will facilitate the choroidal analysis in human myopia research in the future, as it will likely allow more accurate measurements of changes in choroidal thickness compared to the repeatability from the metrological perspective^40,47^.

In conclusion, the present study found variations of repeatability of automated choroidal thickness analysis across the macular area, across subjects and even within the same subject for both OCT devices. As observed, the repeatability improved with increasing eccentricity from the fovea and was found to be better in the nasal regions of the retina. Therefore, upcoming studies with automated choroidal segmentation should focus on the analysis of choroidal thickness changes in the nasal para- and perifoveal retina additionally to the subfovea. Ongoing development in OCT technique, such as swept-source OCT, will allow more precise measurements of choroidal thickness and associated changes in human myopia research in the future.

## Methods

### Subjects

The prospective study adhered to the tenets of the Declaration of Helsinki and was approved by the ethics committee of the Faculty of Medicine of the University Tuebingen. Written informed consent was obtained from all participants. Fifteen subjects aged between 24 years and 37 years with no reported ocular pathologies were enrolled in the study. One subject was excluded as outlier for the agreement comparison as the averaged thickness measurements differed more than 100 *μ*m between both devices.

### OCT devices and scan protocol

Study measurements were performed with two different OCT devices based on spectral-domain technology: ZEISS Cirrus (ZEISS CIRRUS HD-OCT 5000, Carl Zeiss Meditec Inc., Dublin, CA, USA) and Spectralis (HRA + OCT SPECTRALIS, Heidelberg Engineering, Germany). Both devices are able to perform volume scans that consist of multiple B-Scans in a defined retinal area. They are also equipped with an eye-tracking software to minimize motion artifacts during scan acquisition. Moreover, the enhanced depth imaging (EDI) or zero delay method, respectively, was used for a better visualization of the choroid. Other scan settings were also set to match each other as closely as possible. The scan area in both devices covered 6 × 6 mm^2^ for the ZEISS Cirrus and 20 × 20° for the Heidelberg Spectralis, respectively. Furthermore, one B-Scan consisted of 512 A-Scans in both devices, with a frame averaging number of 1 B-Scan. The volume scan for the ZEISS Cirrus consisted of 128 B-Scans, for the Heidelberg Spectralis of 193 B-Scans in total.

Participants underwent nine OCT 3D volume scans with each of the devices on their undilated right eyes. The scans were always obtained by the same examiner. The order of the OCT devices was randomized for each participant. The subjects were instructed to move their head out of and back onto the chin and head rest between the individual scans.

### Choroidal segmentation

Automated choroidal segmentation and thickness analysis was performed with an open-source MATLAB (MATLAB 2017b, The MathWorks, Inc. Natick, MA, USA) software (available online: https://www.mathworks.com/matlabcentral/fileexchange/61275-choroidsegmentation)^34^. Each B-scan of the volume scan was segmented with an resulting matrix as 2D choroidal thickness map. It displays the choroidal thickness of the corresponding retinal location of the scan points with the fovea being assumed in the centre of the scan. Despite the eye tracking software in both OCT devices, the thickness values around the foveola were averaged in the size of a regular microsaccade^48^, in order to obtain the subfoveal choroidal thickness. The rest of the thickness map was divided into the nine ETDRS sections with diameters of 1 mm, 3 mm and 6 mm^37^ and the median was calculated for each of the regions.

### Statistical data analysis

MATLAB and Excel (Microsoft Excel 2016, Microsoft Corporation, Redmond, WA, USA) software was used for the statistical analysis of the data. ANOVA analysis with within-subject standard deviation as conventional repeatability measure was not applicable since the data within the different ETDRS regions did not follow a normal distribution, as tested by the Kolmogorov-Smirnov Test. Nevertheless, to obtain a single value for the repeatability in every ETDRS section, the mean of the nine measurements per subject was subtracted from each of the the nine measurements. The resulting differences to the mean from all subjects per retinal area were then evaluated in a CDF. The 2.5% and 97.5% limits of the cumulative distribution function were identified, multiplied by a correction factor of $\sqrt{3/2}$ for centered data and considered as the limits of the 95% reference interval. Half of the length of the reference interval was then defined as the repeatability value for the analyzed ETDRS region^49^.

To analyze the variability and ranges of intersubject repeatability, the absolute differences to the mean for each subject per ETDRS area were averaged. The standard deviation of the raw choroidal thickness values per subject describe the intrasubject variability of repeatability. These statistical approaches were chosen over the previous methodology using the 95% reference interval, due to the limited statistical and informative value of a CDF with only nine values for each separately analyzed subject. Given that, the values for the intersubject and intrasubject variability of repeatability are lower than the general repeatability value reported for all subjects. To compare the agreement of choroidal thickness analysis for both OCTs, the limits of agreement from the nine averaged choroidal thickness measurements in the different ETDRS sections were calculated via Bland-Altman analysis^38^ and ICC coefficients with ICC(2,k)^50^.

## Data Availability

The datasets generated during and/or analysed during the current study are available from the corresponding author on reasonable request.

## References

[1] Read SA, Fuss JA, Vincent SJ, Collins MJ, Alonso-Caneiro D (2019). Choroidal changes in human myopia: insights from optical coherence tomography imaging. Clin. Exp. Optom..

[2] Wallman J (1995). Moving the retina: choroidal modulation of refractive state. Vis. Res..

[3] Hung L-F, Wallman J, Smith EL (2000). Vision-dependent changes in the choroidal thickness of macaque monkeys. Investig. Ophthalmol. & Vis. Sci..

[4] Zhu X, Park TW, Winawer J, Wallman J (2005). In a matter of minutes, the eye can know which way to grow. Investig. Ophthalmol. & Vis. Sci..

[5] Read SA, Collins MJ, Sander BP (2010). Human optical axial length and defocus. Investig. Ophthalmol. & Vis. Sci..

[6] Chiang ST-H, Phillips JR, Backhouse S (2015). Effect of retinal image defocus on the thickness of the human choroid. Ophthalmic Physiol. Opt..

[7] Wang D (2016). Optical defocus rapidly changes choroidal thickness in schoolchildren. PloS One.

[8] Chiang ST-H, Chen T-L, Phillips JR (2018). Effect of optical defocus on choroidal thickness in healthy adults with presbyopia. Investig. Ophthalmol. & Vis. Sci..

[9] Brown JS (2009). *In vivo* human choroidal thickness measurements: evidence for diurnal fluctuations. Investig. ophthalmology & visual science.

[10] Chakraborty R, Read SA, Collins MJ (2011). Diurnal variations in axial length, choroidal thickness, intraocular pressure, and ocular biometrics. Investig. ophthalmology & visual science.

[11] Tan CS, Ouyang Y, Ruiz H, Sadda SR (2012). Diurnal variation of choroidal thickness in normal, healthy subjects measured by spectral domain optical coherence tomography. Investig. ophthalmology & visual science.

[12] Usui S (2012). Circadian changes in subfoveal choroidal thickness and the relationship with circulatory factors in healthy subjects. Investig. ophthalmology & visual science.

[13] Lee SW, Yu S-Y, Seo KH, Kim ES, Kwak HW (2014). Diurnal variation in choroidal thickness in relation to sex, axial length, and baseline choroidal thickness in healthy korean subjects. Retin..

[14] Chakraborty R (2018). Circadian rhythms, refractive development, and myopia. Ophthalmic Physiol. Opt..

[15] Chakraborty R, Read SA, Collins MJ (2012). Monocular myopic defocus and daily changes in axial length and choroidal thickness of human eyes. Exp. eye research.

[16] Chakraborty R, Read SA, Collins MJ (2013). Hyperopic defocus and diurnal changes in human choroid and axial length. Optom. Vis. Sci..

[17] Deng J (2018). Distribution pattern of choroidal thickness at the posterior pole in chinese children with myopia. Investig. ophthalmology & visual science.

[18] Jin P (2019). Longitudinal changes in choroidal and retinal thicknesses in children with myopic shift. Retin..

[19] Hoseini-Yazdi H, Vincent SJ, Collins MJ, Read SA, Alonso-Caneiro D (2019). Wide-field choroidal thickness in myopes and emmetropes. Sci. reports.

[20] Kong M, Eo DR, Han G, Park SY, Ham D-I (2016). Error rate of automated choroidal segmentation using swept-source optical coherence tomography. Acta Ophthalmol..

[21] Kong M (2018). Measurable range of subfoveal choroidal thickness with conventional spectral domain optical coherence tomography. Transl. Vis. Sci. & Technol..

[22] Rahman W (2011). Repeatability of manual subfoveal choroidal thickness measurements in healthy subjects using the technique of enhanced depth imaging optical coherence tomography. Investig. Ophthalmol. & Vis. Sci..

[23] Branchini L (2012). Reproducibility of choroidal thickness measurements across three spectral domain optical coherence tomography systems. Ophthalmol..

[24] Yamashita T (2012). Repeatability and reproducibility of subfoveal choroidal thickness in normal eyes of japanese using different sd-oct devices. Investig. Ophthalmol. & Vis. Sci..

[25] Shao L (2013). Reproducibility of subfoveal choroidal thickness measurements with enhanced depth imaging by spectraldomain optical coherence tomography. Investig. Ophthalmol. & Vis. Sci..

[26] Li T (2016). Assessment of retinal and choroidal measurements in chinese school-age children with cirrus-hd optical coherence tomography. PloS One.

[27] Alonso-Caneiro D, Read SA, Collins MJ (2013). Automatic segmentation of choroidal thickness in optical coherence tomography. Biomed. Opt. Express.

[28] Chen Q (2015). Automated choroid segmentation based on gradual intensity distance in hd-oct images. Opt. express.

[29] Gupta P (2015). Distribution and determinants of choroidal thickness and volume using automated segmentation software in a population-based study. Am. J. Ophthalmol..

[30] Twa MD, Schulle KL, Chiu SJ, Farsiu S, Berntsen DA (2016). Validation of macular choroidal thickness measurements from automated sd-oct image segmentation. Optom. Vis. Sci..

[31] Maruko I (2010). Subfoveal choroidal thickness after treatment of central serous chorioretinopathy. Ophthalmol..

[32] Maruko I (2011). Subfoveal choroidal thickness after treatment of vogt–koyanagi–harada disease. Retin..

[33] Chung SE, Kang SW, Lee JH, Kim YT (2011). Choroidal thickness in polypoidal choroidal vasculopathy and exudative age-related macular degeneration. Ophthalmol..

[34] Mazzaferri J, Beaton L, Hounye G, Sayah DN, Costantino S (2017). Open-source algorithm for automatic choroid segmentation of oct volume reconstructions. Sci. Reports.

[35] Chen Z, Xue F, Zhou J, Qu X, Zhou X (2016). Effects of orthokeratology on choroidal thickness and axial length. Optom. Vis. Sci..

[36] Breher K, Garcia MG, Ohlendorf A, Wahl S (2018). The effect of the optical design of multifocal contact lenses on choroidal thickness. PloS One.

[37] Group, E. T. D. R. S. R. (1985). Photocoagulation for diabetic macular edema. Arch Ophthalmol.

[38] Bland JM, Altman DG (1999). Measuring agreement in method comparison studies. Stat. Methods Med. Res..

[39] McAlinden C, Khadka J, Pesudovs K (2011). Statistical methods for conducting agreement (comparison of clinical tests) and precision (repeatability or reproducibility) studies in optometry and ophthalmology. Ophthalmic Physiol. Opt..

[40] Mansouri K, Medeiros FA, Tatham AJ, Marchase N, Weinreb RN (2014). Evaluation of retinal and choroidal thickness by swept-source optical coherence tomography: repeatability and assessment of artifacts. Am. J. Ophthalmol..

[41] Hoseini-Yazdi H, Vincent SJ, Collins MJ, Read SA, Alonso-Caneiro D (2019). Repeatability of wide-field choroidal thickness measurements using enhanced-depth imaging optical coherence tomography. Clin. Exp. Optom..

[42] Cho A, Choi Y, Kim Y (2014). Influence of choroidal thickness on subfoveal choroidal thickness measurement repeatability using enhanced depth imaging optical coherence tomography. Eye.

[43] Chen FK (2012). Topographic variation and interocular symmetry of macular choroidal thickness using enhanced depth imaging optical coherence tomography. Investig. Ophthalmol. & Vis. Sci..

[44] Vuong VS (2016). Repeatability of choroidal thickness measurements on enhanced depth imaging optical coherence tomography using different posterior boundaries. Am. J. Ophthalmol..

[45] Zheng F (2016). Choroidal thickness and choroidal vessel density in nonexudative age-related macular degeneration using swept-source optical coherence tomography imaging. Investig. Ophthalmol. & Vis. Sci..

[46] Alonso-Caneiro D, Read SA, Collins MJ (2011). Speckle reduction in optical coherence tomography imaging by affine-motion image registration. J. Biomed. Opt..

[47] Jin P (2016). Choroidal and retinal thickness in children with different refractive status measured by swept-source optical coherence tomography. Am. journal ophthalmology.

[48] Martinez-Conde S, Macknik SL, Troncoso XG, Hubel DH (2009). Microsaccades: a neurophysiological analysis. Trends Neurosci..

[49] ISO/IEC Guide 100:2008. Evaluation of measurement data – Guide to the expression of uncertainty in measurement. Standard, International Organization for Standardization, Geneva, Switzerland (2008).

[50] Shrout PE, Fleiss JL (1979). Intraclass correlations: uses in assessing rater reliability. Psychol. Bull..

